# Management of Type 1 Diabetes in Pregnancy

**DOI:** 10.1007/s11892-016-0765-z

**Published:** 2016-06-24

**Authors:** Anna Z. Feldman, Florence M. Brown

**Affiliations:** Joslin Diabetes Center, 1 Joslin Place, Boston, MA 02115 USA

**Keywords:** Type 1 diabetes, Pregnancy, Preconception, Postpartum

## Abstract

Women with type 1 diabetes (T1DM) have unique needs during the preconception, pregnancy, and postpartum periods. Preconception counseling is essential for women with T1DM to minimize pregnancy risks. The goals of preconception care should be tight glycemic control with a hemoglobin A1c (A1C) < 7 % and as close to 6 % as possible, without significant hypoglycemia. This will lower risks of congenital malformations, preeclampsia, and perinatal mortality. The safety of medications should be assessed prior to conception. Optimal control of retinopathy, hypertension, and nephropathy should be achieved. During pregnancy, the goal A1C is near-normal at <6 %, without excessive hypoglycemia. There is no clear evidence that continuous subcutaneous insulin infusion (CSII) versus multiple daily injections (MDI) is superior in achieving the desired tight glycemic control of T1DM during pregnancy. Data regarding continuous glucose monitoring (CGM) in pregnant women with T1DM is conflicting regarding improved glycemic control. However, a recent CGM study does provide some distinct patterns of glucose levels associated with large for gestational age infants. Frequent eye exams during pregnancy are essential due to risk of progression of retinopathy during pregnancy. Chronic hypertension treatment goals are systolic blood pressure 110–129 mmHg and diastolic blood pressure 65–79 mmHg. Labor and delivery target plasma glucose levels are 80–110 mg/dl, and an insulin drip is recommended to achieve these targets during active labor. Postpartum, insulin doses must be reduced and glucoses closely monitored in women with T1DM because of the enhanced insulin sensitivity after delivery. Breastfeeding is recommended and should be highly encouraged due to maternal benefits including increased insulin sensitivity and weight loss and infant and childhood benefits including reduced prevalence of overweight. In this article, we discuss the care of pregnant patients with T1DM.

## Introduction

Type 1 diabetes (T1DM) affects about 0.1–0.2 % of all pregnancies. Education, effective contraception, preconception planning, tight glycemic control, and comprehensive medical care can decrease maternal, fetal, and pregnancy risks associated with T1DM. Therefore, all women of childbearing age should be counseled about the increased pregnancy risks associated with T1DM to ensure that pregnancies are planned. This review article will discuss the current standards of care and latest research for T1DM and pregnancy in the preconception, pregnancy, and postpartum periods.

## Preconception

### Counseling

**Fig. 1:**
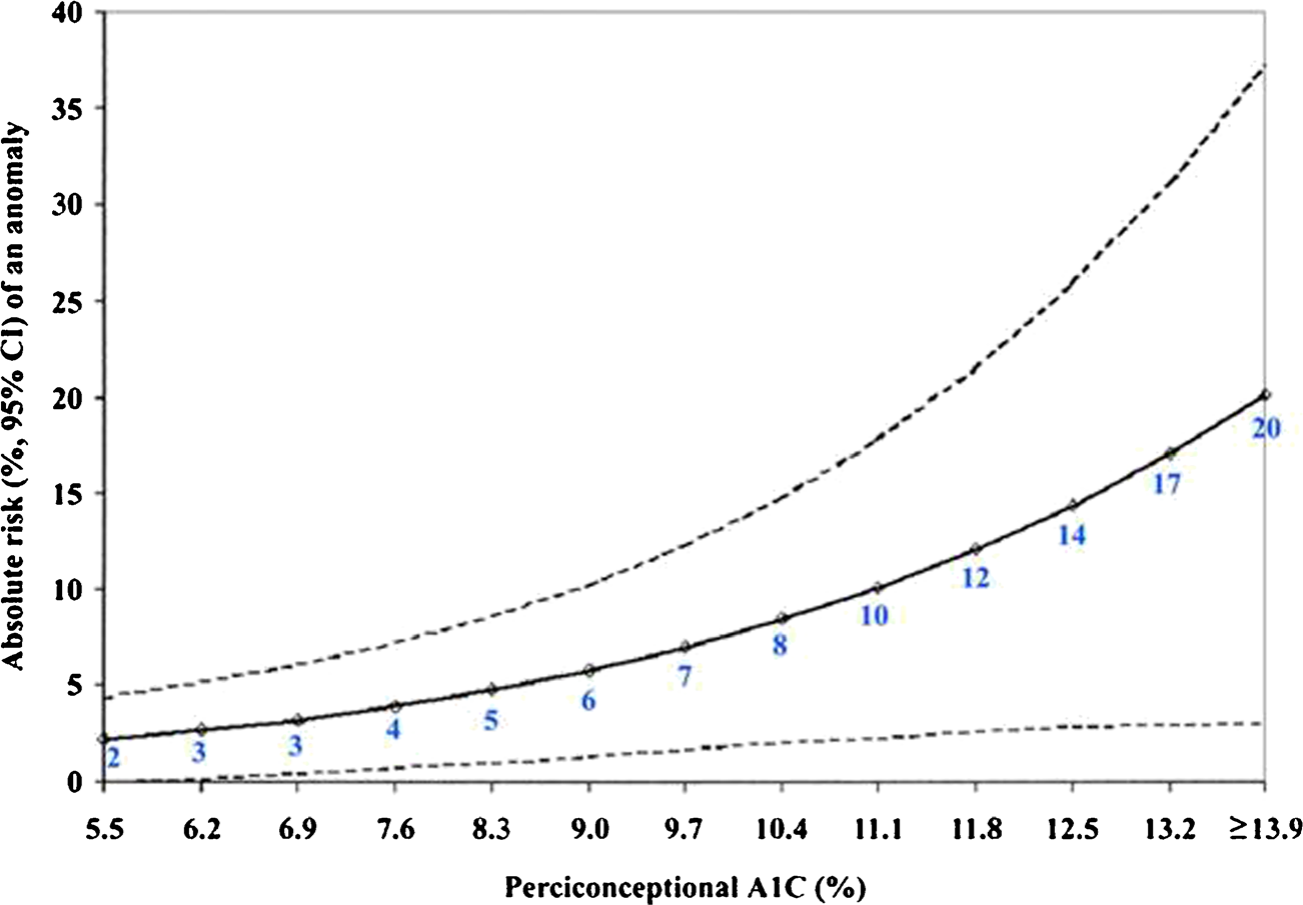
Preconception A1C vs absolute risk of congenital anomaly (with permission from American Diabetes Association: Guerin A, Nisenbaum R, Ray JG. Use of maternal GHb concentration to estimate the risk of congenital anomalies in the offspring of women with prepregnancy diabetes. Diabetes Care 2007;30:1920-5. Copyright and all rights reserved. Material from this publication has been used with the permission of American Diabetes Association) [2]

The goals of preconception care should be tight glycemic control with an A1C <7 % and as close to 6 % as possible without significant hypoglycemia. Since the hemoglobin A1C (A1C) at conception significantly affects pregnancy outcomes, pregnancy planning and preconception counseling regarding tight glycemic control are extremely important for women with T1DM [1]. Elevated blood glucose levels at conception and during the early first trimester are associated with increased rates of congenital malformations, most commonly cardiac and neural tube defects. Compared with the general population rate of 2 %, the prevalence of congenital malformations increases with increasing first trimester A1C (Fig. 1) [2]. Higher A1Cs early in pregnancy are also associated with higher prevalence of spontaneous abortions [3, 4], intrauterine fetal demise [5], preeclampsia [6•], preterm deliveries [7, 8], and perinatal mortality [1].

To improve pregnancy outcomes, preconception care should be comprehensive and include contraception planning, counseling about risks, and optimization of glycemic control, body mass index (BMI), and nutrition. Patients with T1DM who have planned pregnancies enjoy better outcomes, including reduced prevalence of congenital malformations [9], greater gestational age at delivery [10], lower A1C before and during pregnancy [9], lower cesarean delivery rates [11], and decreased perinatal mortality [9]. Tight glycemic control is the cornerstone of preconception care to improve outcomes in this patient population, and stopping contraception only after the goal A1C has been achieved is recommended for best outcomes [12].

Approximately 40–60 % of patients with pre-existing diabetes report that their pregnancies were not planned [13, 14]. Factors associated with planned pregnancies include higher income, higher education levels, private health insurance, endocrinology care prior to pregnancy, married, Caucasian, and encouragement from their physician [14]. Further, physicians often overlook preconception counseling [15]. We recommend ongoing education about contraception and preconception planning starting in the teen years. Contraceptive methods must fit the woman’s lifestyle and be used reliably. Methods include abstinence, barriers, progestin-only and estrogen-progestin pills, injections, implants, patches, vaginal rings, and intrauterine devices. We do not review the advantages and disadvantages of each method in this chapter but do emphasize that for the majority of women, the risks of an unplanned pregnancy greatly outweigh the risks of most forms of contraception. The exception to this is the preference for non-hormonal contraception for women with known increased clotting risk or prior history of venous thrombosis or patent foramen ovale. In general, the intrauterine device is an excellent choice for most women because it is highly effective and is associated with few side effects and does not increase clotting risk. Physicians should outline goals for medical clearance prior to conception and encourage patients to contact them immediately, if they suspect they are pregnant.

### Nutrition

Medical nutrition therapy is needed to optimize glucose management with a focus on consistent timing and quality of healthy meals and snacks and accurate carbohydrate counting. Prenatal vitamins with folic acid reduce the risk of congenital malformations in infants of diabetic mothers [12, 16].

### Diabetes control

Women with T1DM should have monthly visits until control is achieved. After that, medical appointments can be spaced out further. Basal-bolus regimens are beneficial to patients with T1DM [17]. Women with T1DM should begin a basal-bolus regimen preconception, if they are not already on one with either multiple daily injections (MDI) or continuous subcutaneous insulin infusion (CSII) therapy with a goal of achieving target fasting and pre-meal blood glucose and reducing peak postprandial glucose [18]. MDI methods may use either a sliding scale protocol or alternatively match insulin to carbohydrate using a ratio (I:CHO). In pregnancy, superiority of CSII over MDI has not been demonstrated [19, 20]. However, most studies are observational and not randomized controlled trials (RCTs). Optimal A1C levels and timing and targets for self-monitoring of blood glucose (SMBG) are debated. An A1C < 7 % and as close to 6 %, as possible without causing significant hypoglycemia, is recommended, with fasting targets of 80–110 mg/dl and 1-h postmeal targets of 100–155 mg/dl suggested [18, 21].

Insulin generally does not cross the placenta and is thought to be safe in pregnancy. However, the insulin analog glargine has increased affinity for the IGF-1 receptor [22], which plays an important role in fetal development. A recent meta-analysis of studies comparing safety of insulin glargine to NPH insulin concluded that there was no evidence of adverse outcomes related to glargine use in pregnancy [23]. However, long-term risks of glargine in pregnancy are not known, and the FDA has designated glargine as class C. Detemir, another long acting insulin analog, was found to be non-inferior to NPH in an RCT [24, 25•] and is FDA class B in pregnancy. NPH and detemir provide safe and effective basal insulin coverage in pregnancy. For short acting insulin, both aspart and lispro are considered safe in pregnancy [26]. Newer insulin analogs are not well studied.

### Blood Pressure

Preconception blood pressure (BP) goals are now systolic BP < 140 mmHg and diastolic BP < 90 mmHg [27•]. There is strong evidence that ACE-Is and ARBs cause oligohydramnios and renal insufficiency in infants exposed during the second and third trimesters [28]. However, there is some debate about their safety in the first trimester. A study in 2006 suggested that ACE-Is may increase the risk of cardiac and central nervous system malformations in infants exposed in the 1st trimester [29], although recent additional studies on ACE-I and/or ARB exposure during the first trimester of pregnancy have suggested that they are not teratogenic [30–32]. As a result, ACE inhibitors (ACE-Is) and angiotensin receptor blockers (ARBs) may be continued while tight glycemic control is being achieved. The decision to stop or continue them once contraception has been stopped and prior to a positive pregnancy test depends on individual benefits. As such, women with proteinuria, in whom there may be a delay in becoming pregnant, might derive greater benefit from the renal protective effects by continuing them until there is a positive pregnancy test. In most cases, ACE-Is and ARBs should be stopped when clearance to conceive is given. During preconception, BP control can be achieved with methyldopa, calcium channel blockers [33], or the beta blocker labetalol [34•]. Atenolol has been associated with intrauterine growth retardation (IUGR) and is not recommended [35].

### Nephropathy

Kidney disease increases the risk of hypertension, preterm delivery, preeclampsia, IUGR, neonatal jaundice, and mechanical ventilation of the infant. The severity of renal disease is predictive of adverse outcomes, with increasing proteinuria and creatinine inversely associated with gestational age and birth weight [36]. Chronic kidney disease is related to a high risk of both maternal renal failure and perinatal mortality. In women with elevated serum creatinine 1.4–2.7 mg/dl, 43 % had persistent loss of renal function at 6 months postpartum, 59 % of these pregnancies were complicated by preterm delivery, and 37 % had IUGR [37]. Pregnancy does not seem to worsen maternal renal outcomes in women without chronic kidney disease [38]. Preconception assessment should include blood pressure (BP) measurements, serum creatinine, and urine microalbumin.

### Retinopathy

Pregnancy may worsen retinopathy and macular edema [39]. The DCCT showed that diabetic retinopathy was more likely to progress during pregnancy [40]. Risk factors for retinopathy progression during pregnancy include poor glycemic control prior to pregnancy [39–42], rapid improvement of glycemic control [40, 41], previous diabetic retinopathy severity [41], diabetes duration [42], and hypertension [39, 42]. Preconception care should include a dilated retinal exam performed by a retina specialist. Proliferative diabetic retinopathy (PDR) should be treated and quiescent prior to conception.

### Obesity

Obesity is becoming more common in the T1DM patient and is associated with increased risk of intrauterine fetal demise [43], preeclampsia [43, 44], perinatal mortality, and preterm delivery [43]. Therefore, it is imperative to address obesity with lifestyle interventions. Bariatric surgery has not been studied in patients with T1DM who become pregnant. In obese women without diabetes, it has been associated with an increased prevalence of small for gestational age and shorter gestation [45•]. In non-pregnant women with T1DM, gastric bypass reduces weight and BMI at 1 and 5 years, but there is no improvement in A1C levels [46•]. We do not recommend gastric bypass in women with T1DM at this time.

### Thyroid

Autoimmune thyroid disease is common in women with T1DM [47]. Even those patients without known thyroid disease should have a TSH level checked preconception.

### Summary

See preconception checklist (Table 1).

**Table 1:** Preconception checklist

Table 1	Preconception checklist
Achieve optimal glycemic control	HbA1C < 7 % and as close to 6 % as possible without hypoglycemia -Patients should be on basal-bolus regimen with MDI or continuous subcutaneous insulin infusion -Target glucose: fasting 80–110 mg/dl, 1 h postmeal 100–155 mg/dl
Medication assessment	Safety for pregnancy should be assessed -If patient on glargine, switch to detemir -Discontinue statins -Consider stopping ACE-I and ARB in most cases
Medical nutrition counseling	Optimize accuracy of carbohydrate counting for glucose control -Focus on consistent timing and quality of healthy meals
Blood pressure control	Goal <140/90, use an agent acceptable in pregnancy
Dilated retinal exam	Evaluation of retinal status -Treatment and stabilization of proliferative diabetic retinopathy (PDR) before conception
Nephropathy assessment	Check blood pressure (BP) measurements, serum creatinine, and urine microalbumin
Thyroid assessment	Obtain preconception TSH -Goal preconception TSH < 2.5 in patients with known hypothyroidism

## Pregnancy

Pregnant patients with T1DM are complex and require a team approach to their care [48, 49], which should include a diabetologist, obstetrician (perinatal specialist), neonatologist, certified diabetes educator, dietitian, and the patient’s partner. Patients with T1DM have insufficient insulin, causing higher maternal glucose levels. The excess glucose from the maternal circulation results in elevated glucose levels in the fetal circulation, which in turn causes excess fetal adipose growth and increases the risk for large for gestational age (LGA) or macrosomia in infants, especially when the mother is obese [50]. In addition, excessive fuel to the fetus results in increased risk of obesity [51] and impaired glucose tolerance [52, 53] in children of diabetic mothers.

### CSII, MDI, and CGM

Tight glycemic control should be continued throughout the entire pregnancy with an A1C goal <6 % to reduce the occurrence of maternal, fetal, and neonatal complications [18, 54•]. The goal of insulin therapy with either MDI or CSII is to achieve fasting, preprandial and postprandial, and overnight glucose levels in the target range without hypoglycemia. CSII necessitates commitment to frequent glucose monitoring, carbohydrate counting, and rapid implementation of sick day rules in the setting of pump malfunction or illness. Pregnant women with T1DM may benefit from real-time continuous glucose monitoring (CGM), but data on this subject is limited. An RCT did not show that intermittent use of CGM in pregnant women with pre-gestational diabetes improved glycemic control or pregnancy outcomes compared to self-monitoring of plasma glucose multiple times daily [55•]. However, a recent CGM study does provide some distinct patterns of glucose levels associated with large for gestational age infants [56•].

### Insulin Requirements

Insulin requirements vary throughout pregnancy, increasing in the first 9 weeks, decreasing weeks 9–16, and increasing until the 37th week [57]. In the final month of pregnancy, insulin requirements usually decrease [58]. Over the entire pregnancy, insulin requirements may double, and there is a shift toward more prandial insulin relative to basal insulin. [59]. We have observed that insulin delivered right at mealtimes may result in peak postprandial hyperglycemia due to mismatch of glucose absorption into the blood stream and timing of insulin delivery. We recommend giving the mealtime bolus 10–15 min prior to eating if the premeal glucose level is >70 mg/dl and the patient does not have symptoms of hypoglycemia.

Based on the ADA guidelines [18, 60], we recommend fasting glucose targets of 60–99 mg/dl, peak postprandial glucose targets of 100–129 mg/dl, and an A1C of <6 % during pregnancy, without significant hypoglycemia. During labor and delivery, we recommend glucose levels in the 80–110-ng/dl range [61] and use of the insulin drip with D10 [60] to achieve this goal.

### Hypoglycemia

Severe hypoglycemia occurs in up to 50 % of pregnancies in women with T1DM [62], with most occurring in the first trimester [63] and at night [64]. It is most common in women with hypoglycemia unawareness [63]. Hypoglycemia may occur in patients who reactively bolus with correction insulin for an elevated 1 h postprandial blood glucose (bolus stacking) instead of adjusting mealtime bolus insulin (personal obs). Hypoglycemia does not increase the risk of birth defects or fetal death [64], and it does not affect fetal heart fate, breathing, body movement, umbilical artery Doppler wave forms [65], or neonatal intelligence [66]. However, a severe hypoglycemic event could result in a catastrophic event, such as a motor vehicle accident, which could have detrimental effects on the mother [13] and fetus [67]. Pregnant women with T1DM should test glucose levels premeals and postmeals, before and after physical activity, and occasionally in the middle of the night and before driving to monitor for hypoglycemia. CGMs have low glucose alarms which may alert patients of hypoglycemia before the onset of symptoms, especially in those individuals with hypoglycemia unawareness.

### Hyperglycemia

DKA occurs at lower glucose levels because pregnancy is a ketosis-prone state [68, 69]. Factors that may predispose to DKA include infection, insulin omission, pump infusion problems, and use of medications such as terbutaline (to prevent preterm labor) or glucocorticoids (to induce fetal lung maturity) [70–72]. Most are avoidable if close attention is paid to glucose monitoring and sick day rules. Hyperglycemia should be treated promptly with insulin injection by syringe and changing the infusion set and if necessary, presentation to triage for hydration and intravenous insulin and correction of electrolyte abnormalities. For anticipated hyperglycemia when betamethasone is given for fetal lung maturation, Mathiesen et al. describe a useful algorithm for insulin adjustment—also see below “preterm labor” [73]. Neonatal macrosomia, hypoglycemia, and respiratory distress can be decreased when average antepartum glucose levels are <110 mg/dl [61]. Intrapartum glucose levels affect the risk of neonatal hypoglycemia more than antepartum glucose levels; neonatal hypoglycemia risks are lowest when the intrapartum glucose levels are <100 mg/dl [74]. Increased stillbirth rates occur in poorly controlled T1DM [75].

### HTN

For patients who have chronic hypertension on antihypertensives, target blood pressure is systolic blood pressure 110–129 mmHg and diastolic blood pressure 65–79 mmHg [18, 54•, 60]. Goals of therapy for pregnant women with T1DM are derived from data on non-pregnant women with diabetes and pregnant women without diabetes. Hypertension management should ideally start prior to conception, but for women that present for care already pregnant, antihypertensive medications that are safe in pregnancy (described in preconception care) should replace those that are associated with adverse side effects. An RCT of pregnant hypertensive women (not stated as having diabetes) comparing tight (target BP < 140/90 mmHg) vs very tight (target BP < 130/80 mmHg) blood pressure control showed less severe hypertension, fewer hospitalizations, more advanced gestational age, without a difference in preterm delivery or difference in birth weight in the very tight control group [76]. It is important to avoid targeting blood pressure significantly below the mean because of possible increased risk of fetal growth restriction and mortality [77]. Antihypertensives should be initiated to treat chronic hypertension, but not preeclampsia or gestational hypertension [78]. A Cochrane study reviewing RCTs of antihypertensive therapy in pregnancy and a recent RCT demonstrated a halving of the risk of developing severe hypertension with antihypertensive treatment but no improvement in other outcomes [79•, 80•]. Methyldopa appears to be non-inferior to labetalol in preventing adverse outcomes [34•]. Diuretics [81], the beta blocker atenolol and clonidine [35, 82], are generally not used in pregnancy. Fetopathy associated with ACE-Is and ARBs in the second and third trimesters is well established. Severe hypertension may be treated with nifedipine [83•].

### Nephropathy

In uncomplicated T1DM, urinary albumin excretion (UAE) rates are mildly elevated but return to normal by 6 weeks postpartum [84]. Glomelular filtration rate (GFR) increases by 50 % during pregnancy [85]. Patients with diabetic kidney disease may have a marked increase in UAE with nephrotic range proteinuria in some, but in most patients, UAE rates return to baseline levels after delivery [86, 87]. Conversely, patients with diabetic nephropathy with renal insufficiency are at significant risk of GFR decline [88] and renal failure during and after pregnancy (60, see Table 11.19). A retrospective cohort study found increased risk of preterm delivery at 32 weeks in patients with diabetic kidney disease and suboptimal blood pressure control using BP > 130/80 vs <130/80 mmHg (38.1 vs 4.6 %) [84]. Preeclampsia may be difficult to distinguish from worsening diabetic kidney disease and hypertension [78].

### Retinopathy

Diabetic retinopathy (DR) is classified in the same manner in pregnancy as in the non-pregnant population. Pregnancy may cause a temporary worsening of retinopathy [39, 40]. The exact mechanism is uncertain [42, 89]. Known risk factors for progression of retinopathy during pregnancy [60, 90] include diabetes duration, elevated A1C at conception, rapid normalization of glycemic control [41], retinal status at conception (60, see Table 11.22), hypertension, nephropathy, and preeclampsia. Screening eye exams are recommended every trimester during pregnancy, and more frequently if there is significant baseline retinopathy or macular edema. Laser therapy is the treatment of choice based on Diabetic Retinopathy Study [91] and the Early Treatment Diabetic Retinopathy Study [92]. Data on anti-vascular endothelial growth factors (VEGF) exposure in pregnancy is limited to case reports. Theoretically, anti-VEGF mechanisms may be involved in preeclampsia [93] and therefore could negatively impact pregnancy.

### Neuropathy

Diabetes is associated with hyperemesis [94]. Preexisting gastroparesis may worsen during pregnancy, causing severe hyperemesis gravidarum [95]. In a recent RCT of pregnant non-diabetic subjects, ondansetron and metoclopramide both had similar anti-nausea and anti-emetic effects, but ondansetron had fewer side effects and reduced ketonuria but higher cost [96•]. However, a recent review cautions regarding possible increased risk of cardiac congenital malformations with using ondansetron in the first trimester of pregnancy [97•]. Carpal tunnel syndrome is more common in pregnant patients with diabetes [98•]; wrist splints are a non-invasive and safe treatment. There is little data on painful peripheral neuropathy during pregnancy in diabetes, and more data is needed on this subject.

### CV Disease

Only case reports of cardiovascular disease (CVD) in pregnant women with T1DM exist. Age and renal function determine the incidence of CVD in non-pregnant women with T1DM [99•]. Pregnancy may exacerbate pre-existing cardiac disease because it increases cardiac output, heart rate, and hypercoagulability. If a woman has atypical symptoms, ECG, transthoracic echocardiography, or exercise echocardiography should be considered [100]. It may be difficult to discern normal pregnancy symptoms such as dyspnea and fatigue from anginal symptoms. To optimize risk factors, good glycemic control [101], smoking cessation, blood pressure control (targets as above), and lipid management are essential. Dietary modifications to reduce trans and saturated fats may decrease LDL cholesterol. Aspirin therapy is not routinely used for primary prevention in patients with diabetes [102]. The management of myocardial infarction in pregnancy is similar to the non-pregnant state except that ACE-I and ARBs and statins are not used [103, 104].

### Hyperlipidemia

Patients with uncomplicated T1DM who are not pregnant have similar lipid profiles to patients without diabetes [105, 106]. Conversely, patients with T1DM complicated by albuminuria or retinopathy have elevated LDL cholesterol and triglyceride levels compared to patients with uncomplicated T1DM [107]. In pregnant patients, total cholesterol and HDL increase by 50 %, while triglycerides may double; HDL cholesterol increases mid-pregnancy and returns to baseline at the end of pregnancy [108]. Pregnant women with T1DM have similar lipid changes during pregnancy, except that there is less of an increase in HDL mid-gestation [109]. A baseline lipid panel should be obtained at the initial visit if a preconception one is not available [18]. This may help to recognize patients with higher risk of hypertriglyceridemia (those with poorly controlled DM and albuminuria) [107]. Nutrition counseling should be provided on reducing intake of saturated fats to <7 % of caloric intake, elimination of trans fats from the diet, and initiation of exercise walking program if there is no contraindication. In patients with severe hypertriglyceridemia (>1000 mg/dl), omega 3 fatty acids may prevent pancreatitis, although this is rare in the patient with T1DM.

### Thyroid Hypothyroid

Women with Hashimoto’s should have a TSH level every 4–6 weeks during pregnancy. Goal TSH concentrations in pregnancy are 0.1–2.5 μU/ml in the first trimester, 0.2–3 μU/ml in the second trimester, and 0.3–3 μU/ml in the third trimester [110, 111]. The target reference range for total T4 in pregnancy is 1.5 times the normal total T4 due to estrogen-mediated rise in thyroxine-binding globulin in pregnancy [112]. Thyroid hormone replacement requirements rapidly go up between 6 and 16 weeks of gestation, and then plateau after 20 weeks. Usually by 10 weeks, the thyroid hormone dose increases by 30 % compared to baseline; by 20 weeks, the dose is usually increased by 50 % compared to baseline and usually remains stable after [113, 114]. A recommended up titration strategy is to have the patient with hypothyroidism take an extra two pills per week (i.e., nine tabs instead of seven) once they find out they are pregnant until the first prenatal visit [113]. After delivery, thyroid hormone doses usually return to preconception doses.

### Hyperthyroidism

Hyperthyroidism is much less common than hypothyroidism, but it is more common in patients with T1DM than in the general population. Thyroid receptor antibodies (such as TSI or TBII) may help with establishing the diagnosis of Graves’ disease as well as monitoring the disease activity of Graves’ disease who have been previously treated with radioactive iodine or surgery. After delivery, the infant must be monitored for hyperthyroidism with thyroid function tests and heart rate monitoring for tachycardia when the mother has a history of Graves’ disease, even if her disease has been treated. This is because a high thyroid receptor antibody titer in the mother during pregnancy can cause neonatal Graves’ disease. The American Thyroid Association and the American Association of Clinical Endocrinologists recommends using propylthiouracil in the first trimester and methimazole in the second and third trimesters of pregnancy [115]. Radioactive iodine is contraindicated in pregnancy, and thyroidectomy is recommended only in cases where antithyroidal drugs cannot be used.

### LGA

In infants, LGA is defined as weight > 90%ile, while macrosomia is defined as weight > 4000 g at birth. An LGA incidence of 47 % has been noted [116]. The rates of reported macrosomia vary between 29 and 56 % [5, 117, 118]. There is conflicting literature regarding measures of glycemia that predispose to LGA and macrosomia in T1DM pregnancies. Risks associated with LGA/macrosomia include poor glycemic control (A1C > 7 %) in the periconception to 12-week gestation period [119], A1C > 6.5 % in the third trimester [63], elevated fasting [61], and/or preprandial and postprandial blood glucose [120•, 121, 122], elevated average daily blood glucose [123], and episodic elevated postprandial blood glucose despite normal A1C level [124]. Furthermore, increasing A1C > 6 % at 26- and 34-week gestation was associated with increasing prevalence of LGA [120•]. A recent study using CGM data found that women whose infants had LGA had glucose levels that were significantly lower midmorning and early evening in the first trimester, significantly higher in the early morning and throughout the afternoon in the second trimester, and significantly higher during the evening in the third trimester [56•]. We have found that even women who have an A1C < 6 % in the third trimester of pregnancy have LGA prevalence of 25 % (unpublished data, FM. Brown). Thus, it appears that even patients with outstanding diabetes control during pregnancy (A1C < 6 %) have a high risk of having an LGA baby. Other factors that could contribute to LGA may need to be considered to improve this outcome.

### Preterm Delivery

Preterm delivery is birth before 37-week gestation; prevalence varies between 21 and 37 % in T1DM [125] compared to 5.1 % in controls. Risk factors for indicated preterm delivery include A1C > 7 %, worsening nephropathy, preeclampsia, and nulliparity [126]. Increasing levels of third trimester A1C > 6.5 % are associated with increasing prevalence of preterm delivery [120•]. Treatment with antenatal steroids is associated with a decrease in neonatal morbidity and mortality [127]. A recommended algorithm for insulin dosing to control glucose levels after betamethasone 12 mg IM and repeated at 24 h is as follows: increase from baseline total insulin dose of 27, 45, 40, 31, and 11 %, respectively, on days 1–5 from start of steroid therapy [73]. Baby aspirin 81 mg daily is recommended from 12–36 weeks to help reduce risk of preeclampsia in patients with T1DM [128•].

### Summary

See pregnancy checklist (Table 2).

**Table 2:** Pregnancy checklist

Table 2	Pregnancy checklist
Achieve optimal glycemic control	HbA1C < 6 %, or as low as possible without hypoglycemia
Medication assessment	Safety for pregnancy should be assessed -If patient on glargine, switch to detemir -Discontinue statins (ideally preconception) -Stop ACE-Is and ARBs (ideally preconception) -Aspirin 81 mg daily from 12–36 weeks (to help reduce risk of preeclampsia)
Medical nutrition counseling	Optimize accuracy of carbohydrate counting for glucose control -Focus on consistent timing and quality of healthy meals
Blood pressure control for chronic hypertension	Target blood pressure systolic blood pressure 110–129 mmHg and diastolic blood pressure 65–79 mmHg -Use an agent acceptable in pregnancy
Dilated retinal exam	Approximately every trimester or more often if active retinal changes -Laser therapy is treatment of choice for PDR
Nephropathy assessment	Preeclampsia may be difficult to distinguish from worsening diabetic nephropathy and hypertension
Thyroid assessment	Goal TSH first trimester <2.5, 2nd, and 3rd trimester <3 -Increase LT4 dose by 30 % at conception and up to 50 % during pregnancy, usually during first 20 weeks -If patient euthyroid at conception and TPO antibodies positive, monitor TSH every 4–6 weeks during pregnancy In patients with previous history of Graves’ disease, target total T4 is 1.5 times upper range of normal to avoid fetal hypothyroidism. -Propylthiouracil is treatment of choice in first trimester, methimazole is treatment of choice in second and third trimester -Monitor thyroid receptor antibodies if maternal thyroid treated with RAI or surgery to assess for possible fetal exposure

## Postpartum

### Insulin Dosing

After delivery, there is a significant increase in insulin sensitivity; so, a reduction of the dose of insulin to approximately 50 % of the preconception dose is advised. Women who breastfeed will likely have lower basal insulin needs than women who do not breastfeed [129].

### Breastfeeding

Breastfeeding is beneficial for mothers and infants, but women with T1DM may find breastfeeding particularly beneficial due to increased insulin sensitivity [129], weight loss [130], potentially improved sleep if exclusively breastfeeding [131], and improved maternal-fetal bonding. It is unclear if exposure to complex cow’s milk proteins increases the risk of T1DM in susceptible individuals. An intervention trial evaluating a hydrolyzed infant formula to investigate the risk of beta-cell autoimmunity compared with standard formula in women who have discontinued breastfeeding is underway. Early results have not demonstrated a benefit. However, the endpoint will not be reached until 2017 [132•]. Other infant and childhood benefits include reduced prevalence of overweight [133]. Unfortunately, breastfeeding rates in women with T1DM may be lower than in the general population, likely due to both maternal and infant complications [134]. These may include cesarean delivery or a stay in the neonatal intensive care unit resulting in separation of the mother and infant [5, 135], biomechanical issues such as difficulty latching (more common in infants of mothers with T1DM) [136] or delayed lactogenesis [137] which is more common in women with T1DM, or increased episodes of hypoglycemia [129] in the mother. As a result, clinicians treating patients with T1DM in the postpartum period should assess the patient for potential barriers to breastfeeding and provide support to increase rates of initiation and duration of breastfeeding. Ensuring that mothers and infants are not separated when not medically necessary may improve breastfeeding initiation and duration, including early skin to skin contact [138] and night-time feedings [139].

### Medications During Lactation

It is important to take into consideration if a medication has impact on milk production and infant and maternal risks.

### Thyroid

Levothyroxine is FDA-approved for hypothyroid patients during breastfeeding. Antithyroidal medications are safe in breastfeeding in moderate doses (methimazole up to 20–30 mg per day and PTU < 300 mg per day). Methimazole is preferred during breastfeeding over PTU [140]. Infants whose mothers are taking antithyroid medications should have thyroid function testing. Mothers should take their antithyroid drugs immediately after each feeding to reduce infant exposure [111]. Women with T1DM have an increased prevalence of postpartum thyroiditis. Therefore, a TSH level at 3 and 6 months postpartum is recommended in euthyroid women with T1DM [110].

### Lipids

The risk of lipid medications may outweigh the benefits during the short period of lactation. Therefore, these therapies are usually not recommended during breastfeeding. Because atherosclerosis is a chronic condition, stopping these medications for a brief period during lactation should not have a significant effect on long-term cardiovascular disease. The decision to use a lipid lowering agent should be made on a case-by-case basis.

### Contraception

Postpartum contraception with no impact on lactation is preferred [141]. The lactation amenorrhea method or natural family planning with barrier protection is ideal since they do not affect lactation or glucose levels. Intrauterine devices are extremely effective and do not change metabolic status. Progestin-only hormonal contraceptives are a second choice for postpartum contraception and may be administered in pill, implant, or injection form. However, they may lower milk production, particularly if given in the first 6 weeks postpartum. Hormonal contraceptives with both estrogen and progestin usually lower milk production in a dose-dependent manner and are not recommended during lactation.

### Postpartum Weight Retention

Postpartum weight retention is defined as the weight difference between the first year postpartum and the preconception weight; excessive postpartum weight retention is defined as weighing more than 5 kg at 1 year postpartum compared to preconception. Women with T1DM should be encouraged to return to their preconception weight to for optimal glucose control [142•].

## Conclusions

Patients with T1DM who are of reproductive age should be informed about the increased risks associated with pregnancy. Early counseling, pregnancy planning, good glycemic control, and a multi-specialist approach to care before and during pregnancy can all improve pregnancy outcomes for mothers with T1DM and their infants. More research is needed in this patient population, especially in the areas of improving rates of preconception counseling and education, ways to predict and decrease preeclampsia risk, fertility data, treatment of diabetic nephropathy in pregnancy, prevention of infants with LGA and macrosomia, insulin dosing with relation to meal times during pregnancy, medication use during lactation in women with T1DM, and how to improve breastfeeding rates.
